# Clinical symptoms, comorbidities and health outcomes among outpatients infected with the common cold coronaviruses versus influenza virus

**DOI:** 10.1186/s12985-024-02524-6

**Published:** 2024-10-08

**Authors:** Thevambiga Iyadorai, Sin How Lim, Pui Li Wong, Hoe Leong Sii, Chun Keat P’ng, Soon Sean Ee, Maw Pin Tan, Nik Sherina Hanafi, Kim Tien Ng, Jack Bee Chook, Yutaka Takebe, Kok-Gan Chan, Sarbhan Singh, I-Ching Sam, Kok Keng Tee

**Affiliations:** 1 Department of Medical Microbiology, Faculty of Medicine, Universiti Malaya, Kuala Lumpur, Malaysia; 2 Department of Social and Preventive Medicine, Faculty of Medicine, Universiti Malaya, Kuala Lumpur, Malaysia; 3 Department of Medicine, Faculty of Medicine, Universiti Malaya, Kuala Lumpur, Malaysia; 4 Department of Primary Care Medicine, Faculty of Medicine, Universiti Malaya, Kuala Lumpur, Malaysia; 5 Institute of Molecular and Cell Biology, Agency for Science, Technology and Research (A*STAR), Singapore, Singapore; 6 Department of Medical Sciences, School of Medical and Life Sciences, Sunway University, Bandar Sunway, Selangor Darul Ehsan Malaysia; 7 AIDS Research Center, National Institute of Infectious Diseases, Toyama, Shinjuku-ku, Tokyo, Japan; 8 Division of Genetics and Molecular Biology, Institute of Biological Sciences, Faculty of Science, Universiti Malaya, Kuala Lumpur, Malaysia; 9 Jeffrey Cheah School of Medicine and Heath Sciences, Monash University Malaysia, Bandar Sunway, Selangor Darul Ehsan Malaysia; 10 Biomedical Epidemiology Unit, Special Resource Centre, Institute for Medical Research, Ministry of Health, Shah Alam, Malaysia

**Keywords:** Common cold coronaviruses, Influenza virus, Clinical symptom, Comorbidity, Disease burden

## Abstract

**Background:**

Common cold coronaviruses (ccCoVs) and influenza virus are common infectious agents causing upper respiratory tract infections (RTIs). However, clinical symptoms, comorbidities, and health effects of ccCoV infection remain understudied.

**Methods:**

A retrospective study evaluated 3,935 outpatients with acute upper RTI at a tertiary teaching hospital. The presence of ccCoV and influenza virus was determined by multiplex molecular assay. The demographic, clinical symptoms, and health outcomes were compared between patients with ccCoV (*n* = 205) and influenza (*n* = 417) infections. Multivariable logistic regression was employed to evaluate predictors and health outcomes over a one-year follow-up.

**Results:**

Sore throat, nasal discharge, headache, and myalgia were more predominant in ccCoV infection; fever was common in influenza. Most patients reported moderate symptoms severity (49.8% ccCoV, 56.1% influenza). Subsequent primary care visits with symptoms of RTI within a year were comparable for both infections (27.3% ccCoV vs. 27.6% influenza). However, patients with influenza reported increased primary care visits for non-RTI episodes and all-cause hospital admission. Baseline comorbidities were associated with increased primary care visits with symptoms of RTI in either ccCoV (adjusted odds ratio [aOR] 2.5; 95% confidence interval [CI] 1.1–5.9; *P* = 0.034) or influenza (OR 1.9; 95% CI 1.1–3.1; *P* = 0.017) infections, due probably to the dysregulation of the host immune response following acute infections. In patients infected with influenza infection, dyslipidemia was a predictor for subsequent primary care visits with symptoms of RTI (unadjusted OR 1.8; 95% CI 1.0–3.0; *P* = 0.040).

**Conclusions:**

Both influenza and ccCoV infection pose significant disease burden, especially in patients with comorbidities. The management of comorbidities should be prioritized to mitigate poor health outcomes in infected individuals.

**Supplementary Information:**

The online version contains supplementary material available at 10.1186/s12985-024-02524-6.

## Background

In 2019, the global incidence of upper respiratory tract infection (RTI) was estimated to be 17 billion cases [1]. The most common etiological agents responsible for these infections are coronaviruses (CoV), influenza virus, rhinovirus (RV), respiratory syncytial virus (RSV), human metapneumovirus (hMPV), parainfluenza virus (PIV), and adenovirus. Upper RTI affects all populations, particularly young children, older adults, and immunocompromised patients [2]. The coronavirus disease 2019 (COVID-19) pandemic caused by the severe acute respiratory syndrome coronavirus 2 (SARS-CoV-2) further highlights the potential threat of respiratory viruses to global health [3]. Influenza virus has long been recognized and prioritized as a significant threat due to its higher disease burden [2]. However, the clinical and laboratory investigation of other upper RTI-causing viruses are rarely undertaken. This is particularly true in the Southeast Asian region, where routine molecular testing is not widely available [4].

One of the most common types of these upper RTI-causing viruses is CoV. There are two main genera of human CoV: *alphacoronavirus* (229E and NL63) and *betacoronavirus* [OC43, HKU1, severe acute respiratory syndrome coronavirus 1 (SARS-CoV-1), Middle East respiratory syndrome coronavirus (MERS-CoV) and SARS-CoV-2] [5]. Among these human CoV genotypes, 229E, OC43, NL63 and HKU1 are collectively referred to as “common cold coronaviruses” (ccCoV) [5]. Infections caused by the ccCoV strains typically produce mild and self-limiting respiratory symptoms, that in rare instances, may progress to severe respiratory pathologies such as pneumonia and bronchitis [5]. Conversely, in influenza infection, although the symptoms are generally mild, susceptible populations may experience an increase in disease severity and potential complications such as secondary bacterial RTI. The disease severity is expected to be reduced by administration of prophylactic vaccines [6].

Compared to other respiratory viruses, ccCoV infection poses significant challenge in the assessment and evaluation due to the limited understanding of the clinical symptoms, and comorbidities [4]. Although respiratory viruses are typically associated with acute infections that resolve quickly, their potential negative health outcomes cannot be understated [7]. The potential negative health impact of acute ccCoV infection and the associated risk factors also remain largely unknown and unreported.

Understanding the disease burden associated with ccCoV is important in identifying high-risk populations so that they can be prioritized for future antiviral treatments or vaccines. Furthermore, informed clinical decisions can be made in the management of severe diseases caused by ccCoV. This study aims to determine the differences in clinical symptoms, comorbidities, and health outcomes following acute ccCoV or influenza virus infections.

## Methods

### Study population

A retrospective study was conducted at the University Malaya Medical Centre (UMMC), from February 2012 until May 2014, with the sample collection performed during this period. A total of 3,935 outpatients who presented to the hospital’s Primary Care Centre with common cold symptoms, including fever, cough, sore throat, hoarseness of voice, nasal congestion, nasal discharge, sneezing, headache and myalgia were included in this study. Acute upper RTI is defined by the occurrence of these symptoms within the last 14 days. Patients with common cold symptoms for more than 14 days were excluded [8–10].

### Data collection

At the point of recruitment, written informed consent was obtained, and a standardized self-administered questionnaire was used to rate the presence and severity of these common cold symptoms by the patients (except for fever, which was measured and recorded by the attending clinician). Patients were required to rate each of the symptoms as either absent (0), mild (1), moderate (2) or severe (3). Total Symptom Severity Score (TSSS) was tabulated with a possible range of 1–24 points, where a higher score indicates greater severity of respiratory symptoms [11, 12] which can be used as a guideline for the clinicians to determine the suitable type of treatment to be administered.

Demographic information, clinical symptoms, and health outcomes were extracted from their hospital electronic medical records system. The health outcomes evaluated included subsequent walk-in primary care visits due to RTI (upper and/or lower tract) symptoms and non-RTI reasons, all-cause hospitalizations and mortality within a year following ccCoV and influenza virus infections. Primary care visits for RTI reasons are related to respiratory infection-related symptoms, while non-RTI reasons involve non-respiratory infections or other medical issues. RTI-related visits were distinguished from non-RTI visits by identifying symptoms specific to respiratory tract-related infections. All-cause hospitalization includes any admission, regardless of diagnosis while mortality include deaths from any cause. The vital status and date of death information within a year were obtained from the National Registration Department.

To determine the infectious status of the patients at baseline, nasopharyngeal swab samples were collected by clinicians or nurses involved during recruitment. The samples were then transferred to the laboratory in universal transport media and stored at -80 °C until further analysis. The xTAG Respiratory Virus Panel (RVP) FAST multiplex reverse transcription polymerase chain reaction assay (Luminex Molecular Diagnostics, USA) [13] was used to detect the presence of respiratory viruses. A total of 2,008 outpatients enrolled in this study were confirmed to have respiratory viruses. For the subsequent analysis, only ccCoV or influenza virus mono-infections were included as co-infections may present with overlapping and/or synergistic clinical manifestations.

### Statistical analysis

Demographic information, clinical symptoms, and health outcomes were analyzed descriptively using frequencies (N) and percentages (%). The pattern of missing data was examined using statistical software (SPSS, version 26.0) and Little’s missing completely at random (MCAR) test. Based on the Little’s MCAR test, this study had a *P* value of 0.19 which indicated MCAR, as the *P* > 0.05. Because there was < 5% of missing data and the pattern of missingness was MCAR, deletion method was used to address the missingness [14]. This approach aims to minimize the risk of introducing biases and affecting the study’s overall conclusions. For univariate analyses, chi-square test of independence and Fisher’s exact tests were used for independent variables with two categories. Risk factors associated with primary care visits with symptoms of RTI within a year following ccCoV and influenza virus infections were evaluated using binary logistic regression analysis. Multivariate logistic analysis was performed to adjust for potential confounders such as age, gender, and presence of baseline comorbidities. The odds ratio (OR) and 95% confidence interval (CI) were determined and *P* value of < 0.05 was considered statistically significant. Statistical analyses were performed using the Statistical Package for the Social Sciences (SPSS, version 26.0; IBM Corporation).

## Results

### Population characteristics, comorbidities and clinical symptoms

**Fig. 1:**
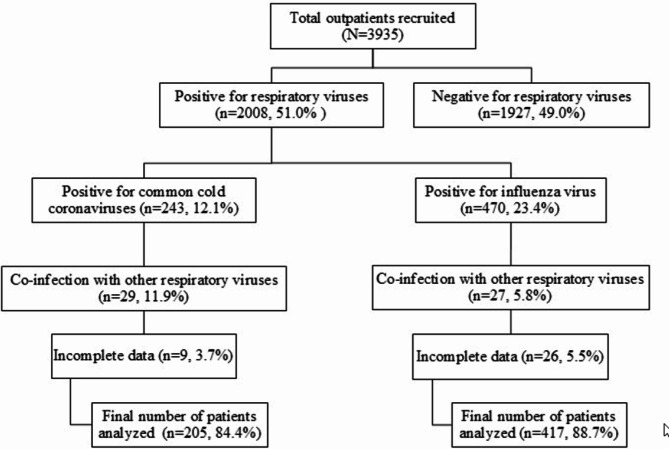
Flowchart depicting test positivity and subsequent inclusion numbers

Among the 3,935 outpatients recruited for this study, 2,008 patients tested positive for the presence of respiratory viruses, with 12.1% (*n* = 243) positive for common cold coronaviruses (ccCoV) [229E (*n* = 37), NL63 (*n* = 59), OC43 (*n* = 72) and HKU1 (*n* = 37)] while 23.4% (*n* = 470) positive for influenza virus [influenza A (*n* = 252) and influenza B (*n* = 165)] **(**Fig. 1**)**. The total patients included in the analysis, after removing co-infections with other respiratory viruses or incomplete data, were 205 and 417 for ccCoV and influenza virus, respectively. According to the demographic characteristics presented in Table 1, there was a higher proportion of females for both infections, with 57.1% in ccCoV cases and 54.2% in influenza cases. The majority of ccCoV infections were among Chinese and Indian patients, each accounting for 33.7%, whereas influenza cases were more common among Malays, representing 42.2% of the cases. The mean age for ccCoV cases was 49.1 years (range, 11–81 years), while for influenza cases, it was 42.1 years (range, 7–91 years). In children (0–18 years), there were 5 cases (2.4%) of ccCoV and 38 cases (9.1%) of influenza infections. Baseline comorbidities were present in 58.5% with ccCoV and 56.1% with influenza **(**Table 1**)**. Comorbidities that were common in ccCoV and influenza cases were hypertension (40.5% vs. 26.4%) (*P* < 0.0001), dyslipidemia (23.9% vs. 17.3%) and diabetes mellitus (18.0% vs. 20.4%). Further analysis on these baseline comorbidities based on different age groups are reported in Additional file 1. In patients with ccCoV, hypertension (68.1%), dyslipidemia (43.6%), diabetes mellitus (30.9%), ischemic heart disease (12.8%) and malignancy (7.4%) were significantly more common in those aged ≥ 55 years. Similarly, in influenza patients, the prevalence of hypertension (60.9%), dyslipidemia (41.4%), diabetes mellitus (46.1%), malignancy (12.5%) and stroke (3.9%) were higher in ≥ 55 years compared to younger patients.

Regarding clinical symptoms reported during recruitment, patients with influenza virus infection had a higher prevalence of fever (83.0%) compared to patients with ccCoV infection (46.8%) (*P* < 0.0001) **(**Table 1**)**. The prevalence of cough was similar in both infections while other clinical symptoms (sore throat, hoarseness of voice, nasal congestion, nasal discharge, sneezing, headache, and myalgia) were more common in ccCoV infection compared to influenza virus infection (*P* < 0.0001). Based on the TSSS for the clinical symptoms, moderate symptoms severity was most common in both groups (49.8% in ccCoV and 56.1% in influenza virus). A detailed analysis of these clinical symptoms across different age groups is provided in Additional file 2. Among the ccCoV patients, those aged 19–54 years had significantly higher rates of fever (58.5%), hoarseness of voice (79.2%), nasal congestion (72.6%) and headache (74.5%) compared to older adults (≥ 55 years). In contrast, for influenza patients, children aged 0–18 years experienced more frequent fever (94.7%), sneezing (28.9%) and headache (23.7%) compared to adults.

**Table 1:** Demographic characteristics, baseline comorbidities, and clinical symptoms of patients with ccCoV and influenza virus infections

Variables	ccCoV	Influenza	*P* value^a^
	*N* = 205 (%)	*N* = 417 (%)	
Gender			0.498
Male	88 (42.9)	191 (45.8)	
Female	117 (57.1)	226 (54.2)	
Ethnicity			0.002*
Malay	62 (30.2)	176 (42.2)	
Chinese	69 (33.7)	87 (20.9)	
Indian	69 (33.7)	136 (32.6)	
Others	5 (2.4)	18 (4.3)	
Age, years, mean (range)	49.1 (11–81)	42.1 (7–91)	< 0.0001*
0–18	5 (2.4)	38 (9.1)	
19–54	106 (51.7)	251 (60.2)	
≥ 55	94 (45.9)	128 (30.7)	
Presence of baseline comorbidities			0.522
None	84 (41.0)	183 (43.9)	
Yes	120 (58.5)	234 (56.1)	
Number of baseline comorbidities			0.838
1	43 (35.8)	88 (37.6)	
2	40 (33.4)	70 (29.9)	
≥ 3	37 (30.8)	76 (32.5)	
Baseline comorbidities			
Hypertension	83 (40.5)	110 (26.4)	< 0.0001*
Dyslipidemia	49 (23.9)	72 (17.3)	0.052
Diabetes mellitus	37 (18.0)	85 (20.4)	0.521
Ischemic heart disease	15 (7.3)	34 (8.2)	0.755
Asthma	12 (5.9)	40 (9.6)	0.126
Malignancy	7 (3.4)	20 (4.8)	0.532
Allergic rhinitis	5 (2.4)	31 (7.4)	0.016*
Other endocrine disease	5 (2.4)	10 (2.4)	1
Chronic renal failure	3 (1.5)	7 (1.7)	1
Chronic obstructive pulmonary disease	2 (1.0)	1 (0.2)	0.252
Obesity	1 (0.5)	16 (3.8)	0.016*
Autoimmune disease	0 (0)	12 (2.9)	0.011*
Smoking status			0.46
Non-smoker	194 (94.6)	392 (94.0)	
Smoker	9 (4.4)	25 (6.0)	
Clinical symptoms			
Fever	96 (46.8)	346 (83.0)	< 0.0001*
Cough	176 (85.9)	353 (84.7)	0.116
Sore throat	148 (72.2)	186 (44.6)	< 0.0001*
Hoarseness of voice	138 (67.3)	16 (3.8)	< 0.0001*
Nasal congestion	124 (60.5)	165 (39.6)	< 0.0001*
Nasal discharge	174 (84.9)	246 (59.0)	< 0.0001*
Sneezing	145 (70.7)	60 (14.4)	< 0.0001*
Headache	116 (56.6)	41 (9.8)	< 0.0001*
Myalgia	118 (57.6)	81 (19.4)	< 0.0001*
Total symptom severity score^b^			0.001*
Mild	83 (40.5)	112 (26.9)	
Moderate	102 (49.8)	234 (56.1)	
Severe	20 (9.8)	71 (17.0)	

Abbreviations: ccCoV, common cold coronaviruses.

^a^*P* value calculated from χ² test or Fisher’s exact test, as appropriate.

^b^ Self-reported symptoms severity for cough, sore throat, hoarseness of voice, nasal congestion, nasal discharge, sneezing, headache, and myalgia.

* *P* < 0.05 is statistically significant.

Unknown/missing data were removed from the analysis

### Health outcomes

Health outcomes were evaluated for one year following the index infection by univariate analysis **(**Table 2**)**. Patients with either ccCoV or influenza virus infections had a similar proportion of primary care visits with symptoms of RTI within one year of the index infections (27.3% and 27.6%, respectively) (patients demographic are reported in Additional file 3). However, the proportion of subsequent primary care visits for non-RTI reasons within a year was higher in patients with influenza virus infection (91.4%) compared to ccCoV infection (43.4%) (*P* < 0.0001). No significant differences were observed for all-cause hospital admission within a year and for one-year mortality following ccCoV or influenza virus index infections **(**Table 2**)**.

**Table 2:** Health outcomes within a year following common cold coronaviruses and influenza virus index infections

Health outcomes	ccCoV	Influenza	*P* value^a^
	*N* = 205 (%)	*N* = 417 (%)	
Primary care visits with symptoms of RTI within a year	56 (27.3)	115 (27.6)	1
Primary care visits for non-RTI reasons within a year	89 (43.4)	381 (91.4)	< 0.0001*
All-cause hospital admission within a year	9 (4.4)	38 (9.1)	0.051
One-year mortality	3 (1.5)	10 (2.4)	0.561

Abbreviations: ccCoV, common cold coronaviruses; RTI, respiratory tract infection.

^a^*P* value calculated from χ² test or Fisher’s exact test, as appropriate.

* *P* < 0.05 is statistically significant.

Unknown/missing data were removed from the analysis

**Fig. 2:**
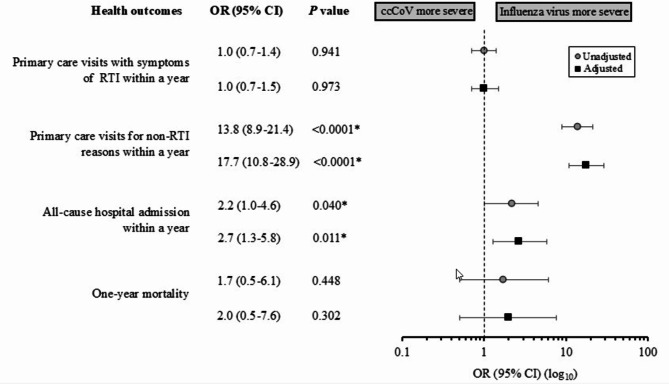
(Un)adjusted regression analysis for health outcomes in patients with ccCoV compared to influenza virus infection. Models were adjusted for gender, age and presence of baseline comorbidities. *P* < 0.05 is considered statistically significant. Abbreviations: ccCoV, common cold coronaviruses; RTI, respiratory tract infection; OR, odds ratio; CI, confidence interval

To assess the health effects in these two patient groups, multivariate logistic regression analysis was conducted **(**Fig. 2**)**. Patients with either ccCoV or influenza virus infections did not differ in terms of their primary care visits with symptoms of RTI within a year, for both unadjusted (OR, 1.0; 95% CI, 0.7–1.4; *P* = 0.941) and adjusted (aOR, 1.0; 95% CI, 0.7–1.5; *P* = 0.973) analyses. However, primary care visits for non-RTI reasons within a year were significantly greater among patients infected with influenza virus compared to those with ccCoV infection. After adjustment for gender, age and the presence of baseline comorbidities, patients infected with influenza virus had 17 times increased odds of primary care visits for non-RTI reasons following their initial presentation (aOR, 17.7; 95% CI, 10.8–28.9; *P* < 0.0001). Moreover, patients with influenza virus infection were 2.7 times more likely to be admitted within a year following the index infection compared to those with ccCoV (aOR, 2.7; 95% CI, 1.3–5.8; *P* = 0.011). Overall, subsequent primary care visits with symptoms of RTI within a year were similar among patients with ccCoV and influenza virus infections, while all-cause hospital admission within a year was higher among patients with influenza virus infection compared to those with ccCoV infection **(**Fig. 2**)**.

The risk factors associated with primary care visits with symptoms of RTI within one year following ccCoV or influenza virus infections were evaluated **(**Table 3**)**. In both cohorts, patients with baseline comorbidities were more likely to have subsequent primary care visits with symptoms of RTI within a year (OR, 2.1; 95% CI, 1.1–4.1; *P* = 0.030 and OR, 1.9; 95% CI, 1.2–2.9; *P =* 0.006, respectively). For patients infected with influenza virus, the number of baseline comorbidities (1, 2 and ≥ 3) and dyslipidemia (OR, 1.8; 95% CI, 1.0–3.0; *P* = 0.040) were significant predictors of primary care visits with symptoms of RTI within a year.

**Table 3:** Unadjusted model of risk factors for primary care visits following ccCoV and influenza virus infections

Variables	Primary care visits with symptoms of RTI					
	ccCoV			Influenza		
	*n* = 56 (%)	OR (95% CI)	*P* value	*n* = 115 (%)	OR (95% CI)	*P* value
Gender						
Male	27 (48.2)	(Ref)		54 (47.0)	(Ref)	
Female	29 (51.8)	0.7 (0.4–1.3)	0.291	61 (53.0)	0.9 (0.6–1.4)	0.771
Age, years, mean (range)	50.2 (16–78)	1.0 (1.0–1.0)	0.501	44.1 (13–80)	1.0 (1.0–1.0)	0.175
Presence of baseline comorbidities						
None	16 (29.1)	(Ref)		38 (33.0)	(Ref)	
Yes	39 (70.9)	2.1 (1.1–4.1)	0.030*	77 (67.0)	1.9 (1.2–2.9)	0.006*
Number of baseline comorbidities						
None	16 (29.1)	(Ref)		38 (33.0)	(Ref)	
1	14 (25.5)	2.3 (1.0-5.3)	0.06	28 (24.3)	1.8 (1.0-3.2)	0.049*
2	13 (23.6)	2.0 (0.9–4.8)	0.109	24 (20.9)	2.0 (1.1–3.7)	0.027*
≥ 3	12 (21.8)	2.0 (0.8–4.8)	0.119	25 (21.7)	1.9 (1.0-3.4)	0.040*
Hypertension						
No	29 (52.7)	(Ref)		80 (69.6)	(Ref)	
Yes	26 (47.3)	1.4 (0.8–2.7)	0.268	35 (30.4)	1.3 (0.8–2.1)	0.247
Dyslipidemia						
No	37 (67.3)	(Ref)		88 (76.5)	(Ref)	
Yes	18 (32.7)	1.8 (0.9–3.6)	0.098	27 (23.5)	1.8 (1.0–3.0)	0.040*
Diabetes mellitus						
No	43 (78.2)	(Ref)		90 (78.3)	(Ref)	
Yes	12 (21.8)	1.4 (0.6–3.1)	0.388	25 (21.7)	1.1 (0.7–1.9)	0.672

Abbreviations: ccCoV, common cold coronaviruses; RTI, respiratory tract infection; OR, odds ratio; CI, confidence interval.

* *P* < 0.05 is statistically significant.

Following adjustment for potential confounders (gender and age), the presence of baseline comorbidities remained a significant predictor of subsequent primary care visits with symptoms of RTI within a year for symptoms of RTI within a year for ccCoV (aOR, 2.5; 95% CI, 1.1–5.9; *P* = 0.034) and influenza virus infection (aOR, 1.9; 95% CI, 1.1–3.1; *P* = 0.017) **(**Table 4**)**. For both cohorts, the presence of baseline comorbidities remained an independent predictor of increased risk of subsequent primary care visits with symptoms of RTI within a year, after adjustment for age, gender, ethnicity, smoking status and TSSS.

**Table 4:** Adjusted model of risk factors for primary care visits following ccCoV and influenza virus infections

Variables	Primary care visits with symptoms of RTI			
	ccCoV		Influenza	
	Adjusted OR	*P* value	Adjusted OR (95% CI)	*P* value
	(95% CI)			
^a^Gender	0.7 (0.4–1.4)	0.34	0.9 (0.6–1.4)	0.744
^a^Age	1.0 (1.0–1.0)	0.499	1.0 (1.0–1.0)	0.983
^a^Presence of baseline comorbidities	2.5 (1.1–5.9)	0.034*	1.9 (1.1–3.1)	0.017*
^b^Hypertension	1.1 (0.4–2.7)	0.913	1.0 (0.5–2.1)	0.892
^b^Dyslipidemia	1.8 (0.8–4.1)	0.176	1.6 (0.9–3.1)	0.124
^b^Diabetes mellitus	1.2 (0.5-3.0)	0.694	0.9 (0.5–1.6)	0.64

Abbreviations: ccCoV, common cold coronaviruses; RTI, respiratory tract infection; OR, odds ratio; CI, confidence interval.

^**a**^ Adjusted for gender, age and presence of baseline comorbidities, as appropriate. Omnibus model coefficient *P* = 0.098 (ccCoV) and *P* = 0.050 (influenza). Hosmer-Lemeshow goodness-of-fit test chi square = 2.875 (df = 8), *P* = 0.942 (ccCoV); chi square = 8.777 (df = 8), *P* = 0.361 (influenza).

^**b**^ Adjusted for gender, age, hypertension, dyslipidemia, and diabetes mellitus, as appropriate. Omnibus model coefficient *P* = 0.553 (ccCoV) and *P* = 0.459 (influenza). Hosmer-Lemeshow goodness-of-fit test chi square = 11.630 (df = 8), *P* = 0.169 (ccCoV); chi square = 7.875 (df = 8), *P* = 0.446 (influenza).

*P < 0.05 is statistically significant

## Discussion

The present study compared the clinical symptoms, baseline comorbidities and risk factors associated with health outcomes of outpatients with ccCoV and influenza virus infections. Our study highlights that fever was more prevalent in influenza-infected patients than those with ccCoV infection. This observation aligns with previous studies comparing influenza virus to other respiratory viruses such as RSV, PIV and hMPV [15, 16]. The higher cases of fever reported in influenza patients might be attributed to increased proinflammatory cytokine and chemokine production [17, 18]. Conversely, the lower cases of fever in ccCoV patients may stem from downregulation of certain proinflammatory cytokines or chemokines, although information on the expression profile of inflammatory markers during acute infection warrants further investigation.

We noted more pronounced upper respiratory tract symptoms in ccCoV patients than in influenza patients. ccCoV exposure can compromise respiratory epithelial cells and their barrier integrity, which in turn may disrupt ciliary pathogen clearance, leading to symptoms like cough and nasal congestion [19, 20]. While influenza can also impair ciliary activity, it does not significantly damage the epithelial barrier, potentially explaining the milder symptomatology [21]. This symptom disparity suggests a potentially higher disease burden and longer recovery for ccCoV infections as compared to influenza virus infection [22].

Our analysis revealed that both ccCoV and influenza virus infections were associated with subsequent primary care visits due to symptoms of RTI. Such visits might result from reinfections or other pathogens [23]. The transient protective immunity post-ccCoV and influenza infections suggest a common immune interference, leading to increased susceptibility to respiratory infection, which may result in more frequent primary care visits within a year [24, 25]. Prior research has associated respiratory infections, like SARS-CoV-2 and RSV, with adverse health outcomes [26, 27]. Our study suggests that ccCoV infections, like influenza, have comparable disease burden. Recognizing this is pivotal for healthcare resource allocation and preventive strategy formulation.

Our analysis shows that the presence of baseline comorbidities was a significant predictor of poor health outcomes. Patients infected with influenza virus were twice as likely to visit primary care within a year. Hypertension and diabetes mellitus have been identified as predictors of adverse health outcomes, including hospitalization, ICU admission or death in influenza and COVID-19 infections [28, 29]. Dysregulated cytokine levels in these patients may compromise innate immune responses, heightening infection risks [30]. Dyslipidemia, in our unadjusted analysis, was linked to increased RTI symptom-related primary care visits in influenza patients. Although the exact role of dyslipidemia in respiratory viral infections remains elusive, elevated IL-10 levels in these patients might weaken T cell-mediated infection defenses [31–33]. In essence, our findings emphasize the significant health repercussions of both ccCoV and influenza virus infections, underscoring the need for vigilant monitoring and intervention.

The cohort in this study was recruited prior to the COVID-19 pandemic, making it highly appropriate for evaluating host and viral factors, as well as the risks associated with disease pathogenesis, such as symptom severity and health outcomes in ccCoV-infected patients. Such inclusion criteria are essential to mitigate the effect of potential cross-protective immunity developed following SARS-CoV-2 infection or COVID-19 vaccination that can confer protection against other ccCoV. Studies have shown that SARS-CoV-2 infection [34, 35] and vaccination [36–38] can confer broad protection against heterologous coronaviruses, including ccCoV, with the S2 subdomain of the spike protein of coronaviruses being the main target for cross-reactivity. To minimize the effect of cross-protective immunity from SARS-CoV-2 infection and/or vaccination on the pathogenesis of ccCoV infections, which includes symptom severity during acute infection and health outcomes in infected patients, a study population recruited prior to the COVID-19 pandemic is essential to achieve the study objective with higher degree of confidence.

This study was limited by its single-centre recruitment within the urban setting, therefore, potentially reducing its generalizability to other populations. Second, this study did not investigate the infection status of patients during subsequent visits with symptoms of RTI, which could potentially be caused by other pathogens. Therefore, the effect of viral or bacterial interference during ccCoV and influenza virus infections affecting health outcomes could not be determined [39].

## Conclusion

In conclusion, our findings delineate the clinical and health outcomes of ccCoV and influenza infections, emphasizing the need for further research. The data suggests prioritizing comorbidity and dyslipidemia management to reduce the risk of poor health outcomes in infected individuals. The study also underscores the need for increased emphasis on RTI diagnostics, treatment options such as antiviral drugs and vaccination, and preventive measures to mitigate the impact of respiratory infections on public health.

## Electronic supplementary material

Below is the link to the electronic supplementary material.

Supplementary Material 1

Supplementary Material 2

Supplementary Material 3

## Data Availability

No datasets were generated or analysed during the current study.

## Abbreviations

ccCoV: Common cold coronaviruses
RTI: Respiratory tract infection
OR: Odds ratio
aOR: Adjusted odds ratio
CI: Confidence interval
RV: Rhinovirus
RSV: Respiratory syncytial virus
hMPV: Human metapneumovirus
PIV: Parainfluenza virus
COVID-19: Coronavirus disease 2019
SARS-CoV: Severe acute respiratory syndrome coronavirus
MERS-CoV: Middle East respiratory syndrome coronavirus
UMMC: Universiti Malaya Medical Centre
TSSS: Total symptom severity score
ICU: Intensive care unit
IL: Interleukin
