# Supplementary material for: Clinical symptoms, comorbidities and health outcomes among outpatients infected with the common cold coronaviruses versus influenza virus
Source: Virol J. 2024 Oct 8;21:251. doi: 10.1186/s12985-024-02524-6 (PMC11462790; doi:10.1186/s12985-024-02524-6)
Supplement: Supplementary file 1 — Supplementary Material 1 [file 12985_2024_2524_MOESM1_ESM.docx]

**Additional file 1.** Baseline comorbidities of patients with common cold coronaviruses and influenza virus infections.

| Baseline comorbidities | ccCoV  N=205 (%) | | | | Influenza  N=417 (%) | | | |
| --- | --- | --- | --- | --- | --- | --- | --- | --- |
|  | 0-18 years  n=5 (%) | 19-54 years  n=106 (%) | ≥ 55 years  n=94 (%) | *P* value^a^ | 0-18 years  n=38 (%) | 19-54 years  n=251 (%) | ≥ 55 years  n=128 (%) | *P* value^a^ |
| Hypertension  Dyslipidemia  Diabetes mellitus  Ischemic heart disease  Asthma  Malignancy  Allergic rhinitis  Other endocrine disease  Chronic renal failure  Chronic obstructive pulmonary disease  Obesity  Autoimmune disease  Depression  Benign prostatic hyperplasia  Osteoarthritis  Schizophrenia  Chronic liver disease  Gastritis  Osteoporosis  Stroke  Parkinson’s disease  Rheumatic heart disease  Thalassemia  Cervical spondylosis  Gastroesophageal reflux disease  Glaucoma  Immune thrombocytopenia  Rheumatoid arthritis  Valvular heart disease  Pulmonary tuberculosis  Viral hepatitis  Epilepsy  Sleep apnoea  Glucose-6-phosphate dehydrogenase  Immunodeficiency  Anaemia  Alzheimer’s disease  Ankylosing spondylitis  Marfan syndrome  Paroxysmal atrial fibrillation  Polycystic ovary syndrome  Scoliosis  Spina bifida | 0 (0.0)  0 (0.0)  0 (0.0)  0 (0.0)  1 (20.0)  0 (0.0)  0 (0.0)  0 (0.0)  0 (0.0)  0 (0.0)  0 (0.0)  0 (0.0)  0 (0.0)  0 (0.0)  0 (0.0)  0 (0.0)  0 (0.0)  0 (0.0)  0 (0.0)  0 (0.0)  0 (0.0)  0 (0.0)  0 (0.0)  0 (0.0)  0 (0.0)  0 (0.0)  0 (0.0)  0 (0.0)  0 (0.0)  0 (0.0)  0 (0.0)  0 (0.0)  0 (0.0)  0 (0.0)  0 (0.0)  0 (0.0)  0 (0.0)  0 (0.0)  0 (0.0)  0 (0.0)  0 (0.0)  0 (0.0)  0 (0.0) | 19 (18.0)  8 (7.5)  8 (7.5)  3 (2.8)  9 (8.4)  0 (0.0)  3 (2.8)  1 (0.9)  0 (0.0)  0 (0.0)  1 (0.9)  0 (0.0)  3 (2.8)  0 (0.0)  0 (0.0)  1 (0.9)  1 (0.9)  1 (0.9)  0 (0.0)  0 (0.0)  0 (0.0)  1 (0.9)  1 (0.9)  0 (0.0)  1 (0.9)  1 (0.9)  1 (0.9)  0 (0.0)  1 (0.9)  0 (0.0)  0 (0.0)  0 (0.0)  0 (0.0)  0 (0.0)  0 (0.0)  0 (0.0)  0 (0.0)  0 (0.0)  0 (0.0)  0 (0.0)  0 (0.0)  0 (0.0)  0 (0.0) | 64 (68.1)  41 (43.6)  29 (30.9)  12 (12.8)  2 (2.1)  7 (7.4)  2 (2.1)  4 (4.3)  3 (3.2)  2 (2.1)  0 (0.0)  0 (0.0)  4 (4.3)  3 (3.2)  3 (3.2)  1 (1.1)  1 (1.1)  1 (1.1)  2 (2.1)  1 (1.1)  1 (1.1)  0 (0.0)  0 (0.0)  1 (1.1)  0 (0.0)  0 (0.0)  0 (0.0)  1 (1.1)  0 (0.0)  0 (0.0)  0 (0.0)  0 (0.0)  0 (0.0)  0 (0.0)  0 (0.0)  0 (0.0)  0 (0.0)  0 (0.0)  0 (0.0)  0 (0.0)  0 (0.0)  0 (0.0)  0 (0.0) | < 0.0001*  < 0.0001*  < 0.0001*  0.025*  0.054  0.018*  1.000  0.286  0.168  0.260  1.000  NA  0.755  0.168  0.168  1.000  1.000  1.000  0.260  1.000  1.000  1.000  1.000  1.000  1.000  1.000  1.000  1.000  1.000  NA  NA  NA  NA  NA  NA  NA  NA  NA  NA  NA  NA  NA  NA | 0 (0.0)  0 (0.0)  0 (0.0)  1 (2.6)  3 (7.9)  0 (0.0)  3 (7.9)  1 (2.6)  0 (0.0)  0 (0.0)  0 (0.0)  0 (0.0)  1 (2.6)  0 (0.0)  0 (0.0)  0 (0.0)  0 (0.0)  0 (0.0)  0 (0.0)  0 (0.0)  0 (0.0)  0 (0.0)  1 (2.6)  0 (0.0)  0 (0.0)  0 (0.0)  0 (0.0)  0 (0.0)  0 (0.0)  0 (0.0)  0 (0.0)  0 (0.0)  1 (2.6)  1 (2.6)  0 (0.0)  0 (0.0)  0 (0.0)  0 (0.0)  0 (0.0)  0 (0.0)  0 (0.0)  0 (0.0)  0 (0.0) | 32 (12.7)  19 (7.6)  26 (10.4)  17 (6.8)  28 (11.2)  4 (1.6)  20 (8.0)  5 (2.0)  2 (0.8)  1 (0.4)  10 (4.0)  10 (4.0)  4 (1.6)  0 (0.0)  0 (0.0)  2 (0.8)  0 (0.0)  0 (0.0)  0 (0.0)  0 (0.0)  0 (0.0)  1 (0.4)  0 (0.0)  0 (0.0)  0 (0.0)  0 (0.0)  0 (0.0)  0 (0.0)  0 (0.0)  6 (2.4)  3 (1.2)  3 (1.2)  1 (0.4)  1 (0.4)  1 (0.4)  1 (0.4)  0 (0.0)  1 (0.4)  1 (0.4)  0 (0.0)  1 (0.4)  1 (0.4)  1 (0.4) | 78 (60.9)  53 (41.4)  59 (46.1)  16 (12.5)  9 (7.0)  16 (12.5)  8 (6.3)  4 (3.1)  5 (3.9)  0 (0.0)  6 (4.7)  2 (1.6)  3 (2.3)  0 (0.0)  0 (0.0)  1 (0.8)  0 (0.0)  0 (0.0)  0 (0.0)  5 (3.9)  2 (1.6)  0 (0.0)  0 (0.0)  0 (0.0)  0 (0.0)  0 (0.0)  0 (0.0)  0 (0.0)  0 (0.0)  3 (2.3)  5 (3.9)  2 (1.6)  2 (1.6)  0 (0.0)  1 (0.8)  1 (0.8)  1 (0.8)  0 (0.0)  0 (0.0)  1 (0.8)  0 (0.0)  0 (0.0)  0 (0.0) | < 0.0001*  < 0.0001*  < 0.0001*  0.087  0.458  < 0.0001*  0.844  0.614  0.086  1.000  0.500  0.339  0.394  NA  NA  1.000  NA  NA  NA  0.006*  0.158  1.000  1.000  NA  NA  NA  NA  NA  NA  1.000  0.178  1.000  0.125  0.268  1.000  1.000  1.000  1.000  1.000  1.000  1.000  1.000  1.000 |

Abbreviations: ccCoV, common cold coronaviruses.

^a^ *P* value calculated from χ² test or Fisher’s exact test, as appropriate.

* *P* <0.05 is statistically significant.
