# Supplementary material for: Clinical symptoms, comorbidities and health outcomes among outpatients infected with the common cold coronaviruses versus influenza virus
Source: Virol J. 2024 Oct 8;21:251. doi: 10.1186/s12985-024-02524-6 (PMC11462790; doi:10.1186/s12985-024-02524-6)
Supplement: Supplementary file 2 — Supplementary Material 2 [file 12985_2024_2524_MOESM2_ESM.docx]

**Additional file 2.** Clinical symptoms of patients with common cold coronaviruses and influenza virus infections.

| Clinical symptoms | ccCoV  N=205 (%) | | | | Influenza  N=417 (%) | | | |
| --- | --- | --- | --- | --- | --- | --- | --- | --- |
|  | 0-18 years  n=5 (%) | 19-54 years  n=106 (%) | ≥ 55 years  n=94 (%) | *P* value^a^ | 0-18 years  n=38 (%) | 19-54 years  n=251 (%) | ≥ 55 years  n=128 (%) | *P* value^a^ |
| Fever  Cough  Sore throat  Hoarseness of voice  Nasal congestion  Nasal discharge  Sneezing  Headache  Myalgia | 3 (60.0)  4 (80.0)  4 (80.0)  2 (40.0)  3 (60.0)  3 (60.0)  3 (60.0)  3 (60.0)  2 (40.0) | 62 (58.5)  92 (86.8)  83 (78.3)  84 (79.2)  77 (72.6)  93 (90.6)  84 (79.2)  79 (74.5)  70 (66.0) | 31 (33.0)  80 (85.1)  61 (64.9)  52 (55.3)  44 (46.8)  75 (79.8)  58 (61.7)  34 (36.2)  46 (48.9) | 0.005*  0.633  0.276  0.002*  0.004*  0.064  0.062  <0.0001*  0.102 | 36 (94.7)  31 (81.6)  18 (47.4)  2 (5.3)  17 (44.7)  26 (68.4)  11 (28.9)  9 (23.7)  8 (21.1) | 216 (86.1)  206 (82.1)  116 (46.2)  10 (4.0)  96 (38.2)  149 (59.4)  39 (15.5)  29 (11.6)  51 (20.3) | 94 (73.4)  116 (90.6)  52 (40.6)  4 (3.1)  52 (40.6)  71 (55.5)  10 (7.8)  3 (2.3)  22 (17.2) | 0.001*  0.078  0.541  0.732  0.693  0.358  0.004*  <0.0001*  0.743 |

Abbreviations: ccCoV, common cold coronaviruses.

^a^ *P* value calculated from χ² test or Fisher’s exact test, as appropriate.

* *P* <0.05 is statistically significant.
