# Supplementary material for: Clinical symptoms, comorbidities and health outcomes among outpatients infected with the common cold coronaviruses versus influenza virus
Source: Virol J. 2024 Oct 8;21:251. doi: 10.1186/s12985-024-02524-6 (PMC11462790; doi:10.1186/s12985-024-02524-6)
Supplement: Supplementary file 3 — Supplementary Material 3 [file 12985_2024_2524_MOESM3_ESM.docx]

| Variables | ccCoV  n=56 (%) | Influenza  n=115 (%) |
| --- | --- | --- |
| Gender  Male  Female  Ethnicity  Malay  Chinese  Indian  Others  Age, years  0-18  19-54  ≥ 55  Presence of baseline comorbidities  None  Yes  Number of baseline comorbidities  1  2  ≥ 3  Baseline comorbidities  Hypertension  Dyslipidemia  Diabetes mellitus  Ischemic heart disease  Asthma  Malignancy  Allergic rhinitis  Other endocrine disease  Chronic renal failure  Chronic obstructive pulmonary disease  Obesity  Autoimmune disease  Smoking status  Non-smoker  Smoker | 27 (48.2)  29 (51.8)  18 (32.1)  19 (33.9)  19 (33.9)  0 (0.0)  1 (1.8)  29 (51.8)  26 (46.4)  16 (29.1)  39 (70.9)  14 (25.5)  13 (23.6)  12 (21.8)  26 (47.3)  18 (32.7)  12 (21.8)  3 (5.5)  3 (5.5)  0 (0.0)  2 (3.6)  4 (7.3)  0 (0.0)  1 (1.8)  0 (0.0)  0 (0)  54 (98.2)  1 (1.8) | 54 (47.0)  61 (53.0)  51 (44.3)  27 (23.5)  36 (31.3)  1 (0.9)  11 (9.6)  66 (57.4)  38 (33.0)  38 (33.0)  77 (67.0)  28 (24.3)  24 (20.9)  25 (21.7)  35 (30.4)  27 (23.5)  25 (21.7)  13 (11.3)  14 (12.2)  5 (4.3)  13 (11.3)  5 (4.3)  3 (2.6)  0 (0.0)  5 (4.3)  5 (4.3)  106 (92.2)  9 (7.8) |

**Additional file 3.** Demographic characteristics of patients who visited primary care for RTI symptoms within a year following common cold coronaviruses and influenza virus infections.

Abbreviations: ccCoV, common cold coronaviruses.
